# Assessing the role of family well-being on the quality of life of Indian children with thalassemia

**DOI:** 10.1186/s12887-019-1466-y

**Published:** 2019-04-08

**Authors:** Arulmani Thiyagarajan, M Bagavandas, Kalpana Kosalram

**Affiliations:** School of Public Health, SRM University, Chennai, India

**Keywords:** Health-related quality of life, Thalassemia, Psychological well-being, Family income, Parent’s education

## Abstract

**Background:**

The association between chronic diseases and psychological problems is well established. As thalassemia is chronic blood disorder with burdensome treatment procedures, patients are likely to have psychological health problems. Many studies reported evidences regarding the quality of life. But, factors influencing the health-related quality of life with focus on psychological well-being were minimally studied. We aimed to find the factors contributing to the health-related quality of life among thalassemia affected children and hypothesising whether the parent’s psychological well-being, sociodemographic characteristics and transfusion interval have an impact on children’s quality of life.

**Method:**

A cross-sectional analytical study conducted on 125 thalassemia patients and 125 parents (either father or mother) referred to the clinic of Thalassemia treatment center. KIDSCREEN-10 and Ryff Psychological well-being scale is used for measuring the health-related quality of life and well-being of children and parent respectively.

**Results:**

We have found the three factors such as family income, children education, and, parent education significantly contributed to the children’s health-related quality of life among thalassemia affected children. The average score of Health-related quality of life among children is 16.28 with a standard deviation of 3.432 and the mean psychological well-being score for the parent is 83.99 with a standard deviation of 11.41. A positive correlation exists between parent psychological well-being and children’s health-related quality of life.

**Conclusion:**

Family well-being is the foundation for quality of life of the children. It was found that factors such as family income and parents’ and children’s education have a direct association with HRQoL of life of children with thalassemia. However, more studies need to be done in order to ascertain the factors contributing to HRQoL of children with thalassemia to improve the quality of life of thalassemia patients.

## Background

Study of the relationship between disease and the sense of emotion has always been an interesting area of research. The associations between chronic disease conditions and psychological problems is well established [1, 2]. the topics of psychology and concentration of well-being have grown popular in ambit of public policy [3, 4]. This has led to an interest in a less researched aspect of the relationship between emotion and disease, namely, the impact of disease condition and well-being.

Thalassemia is a chronic disorder. It is a life-threatening and life-limiting condition that affects the patient clinically and psychologically by its burdensome treatment process: regular blood transfusions, iron chelation, frequent hospitalization and medical follow-up [5]. Globally, the prevalence of thalassemia ranges between 2 and 25% [6]. For every 100,000 live births, approximately 4.4 children are affected by thalassemia throughout the world [7]. Disease burden also increases because of repeated visits to the hospital, repeated laboratory tests and frequent monitoring of symptoms in detecting complications [8]. The paucity of healthcare policies, inadequate treatment support and lack of regular screening contribute to an increase in vulnerability to the disease. India likely to have a higher burden as there are no health policies or preventive checks like in other countries (Cyprus, Iran, Pakistan, Palestine territories) [9, 10]. Every year, 10,000 children are born with thalassemia, which approximately accounts for 10% of the world’s total incidence of thalassemia and one in eight of thalassemia patients’ lives in India [11].

Majority of thalassemia patients suffer from depressive symptoms and mental disorders [12–14]. Children, teenagers, and families that have patients with thalassemia are more susceptible to facing emotional and behavioral problems. The burden of thalassemia challenges the entire family at physical, cognitive, and emotional levels and disrupts their life as a whole [15]. The recurring and complex treatment procedure often places undue psychological and financial burden on the individual and the family.

Researchers have documented the status of well-being, health-related quality of life and burden in families that have children with thalassemia. There is lack of evidence in regard to factors contributing to their quality of life. For improving the quality of life of thalassemia patients, it is imperative to understand the factors that contribute to it.

In this research, we aim to assess the factors influencing the health-related quality of life and also hypothesise whether the parent’s psychological well-being has an impact on children’s quality of life. It could possibly pave the way to understand the area that needs to be focused upon for improving the quality of life among thalassemia patients.

## Objectives

1. To assess the factors influencing the health-related quality of life.

2. To hypothesise whether the parent’s psychological well-being, sociodemographic characteristics and transfusion interval have an impact on children’s quality of life.

## Methods

### Study design

A cross-sectional analytical study conducted on 125 thalassemia patients (along with either one of their parents) referred to a thalassemia treatment centre. The study was conducted from 5 January to 31 July 2017.

### Study setting

The treatment center is a Voluntary Health Services, which has a separate unit for treating thalassemia patients and patients with other blood disorders. It is situated in a very prominent locality in Chennai and has registered patients from the different parts of India.

### Study population and study size

The inclusion criteria for the study were patients who had transfusion-dependent (major) thalassemia and were less than 18 years of age. Children suffering from debilitating disorders other than thalassemia major were excluded from the study. Similarly, 125 parents (either mother or father) of the children were included in the study. The sample size was calculated using the formula $$ \raisebox{1ex}{${z}^2 pq$}\!\left/ \!\raisebox{-1ex}{${d}^2$}\right. $$ (95% C. I, Prevalence – 4%, Precision – 6%) [16]. We performed a complete enumeration of patients who visited thalassemia treatment centre during our study period.

### Instrument

#### Kidscreen-10

The KIDSCREEN-10 is a questionnaire developed and normalised for surveying health-related quality of life (HRQoL) in children and adolescents. Existing validation results provided a single- dimensionality HRQoL index consisting of 10 items, which sufficiently represents the longer KIDSCREEN profiles [17]. We used three-point rating scale (0, 1, and 2) with the indicator being the better the score, better the HRQoL.

#### Ryff psychological well-being scale (RPWBS)

Carol Ryff has conceptualised psychological well-being through a questionnaire consisting of six dimensions: autonomy, environmental mastery, personal growth, positive relations with others, purpose in life, self-acceptance [18]. Parent’s well-being score is the total of all the components mentioned. We considered the Ryff scale to capture the psychological well-being among parents of thalassemia-affected children, focusing on the psychological component of well-being as the disease may have a psychological impact at the family level [19]. Validated versions of the tools were used for data collection. We used three-point rating scale (0,1,2), with the indicator being better the score, better their well-being.

### Variables and data collection, data analysis and statistics

#### Source of data and collection

Data were collected from the individual parent and children through the questionnaires after getting their approval to participate. Personally identifiable information like name, and address was not recorded keeping the data anonymous. Variables collected from the questionnaires include the sociodemographic characteristics, disease features, and questions comprising for HRQoL among children and questions related to psychological well-being of parents. We used Microsoft Excel for data entry and Statistical Package for Social Sciences (SPSS) version 23 for statistical analysis. Data were double entered and all inconsistencies were resolved using the original data collection sheet.

### Statistical analysis

Data were first entered in an Excel sheet and then transferred to the SPSS version 23. On preliminary analysis, observations containing incomplete questionnaires were removed and thoroughly checked for errors. Data were normally distributed. Socio-demographic characteristics, disease features, and HRQoL components and psychological well-being components were analysed. We used the *t*-test to compare the quality of life of male and female children and the well-being of the mother and father. Pearson correlation coefficient was used to assess the relationship between scores of HRQoL and psychological well-being scores of parents. Multiple linear regression analysis was performed to assess the factors contributing to children’s HRQoL.

### Ethical considerations

The Institutional Review Board (IRB) and the research ethics committee of the School of Public Health, SRM Institute of Science and Technology approved the study. Due permissions were received from relevant authorities in thalassemia treatment center at Voluntary Health Services. All the participants in the study were informed about the study objectives and signed a written informed consent form and were assured of the confidentiality of their personal information. The participants were also informed that the data obtained from them would be used for publication. However, their personal identifiers would be kept anonymous and confidentiality of their personal records maintained.

## Results

**Fig. 1:**
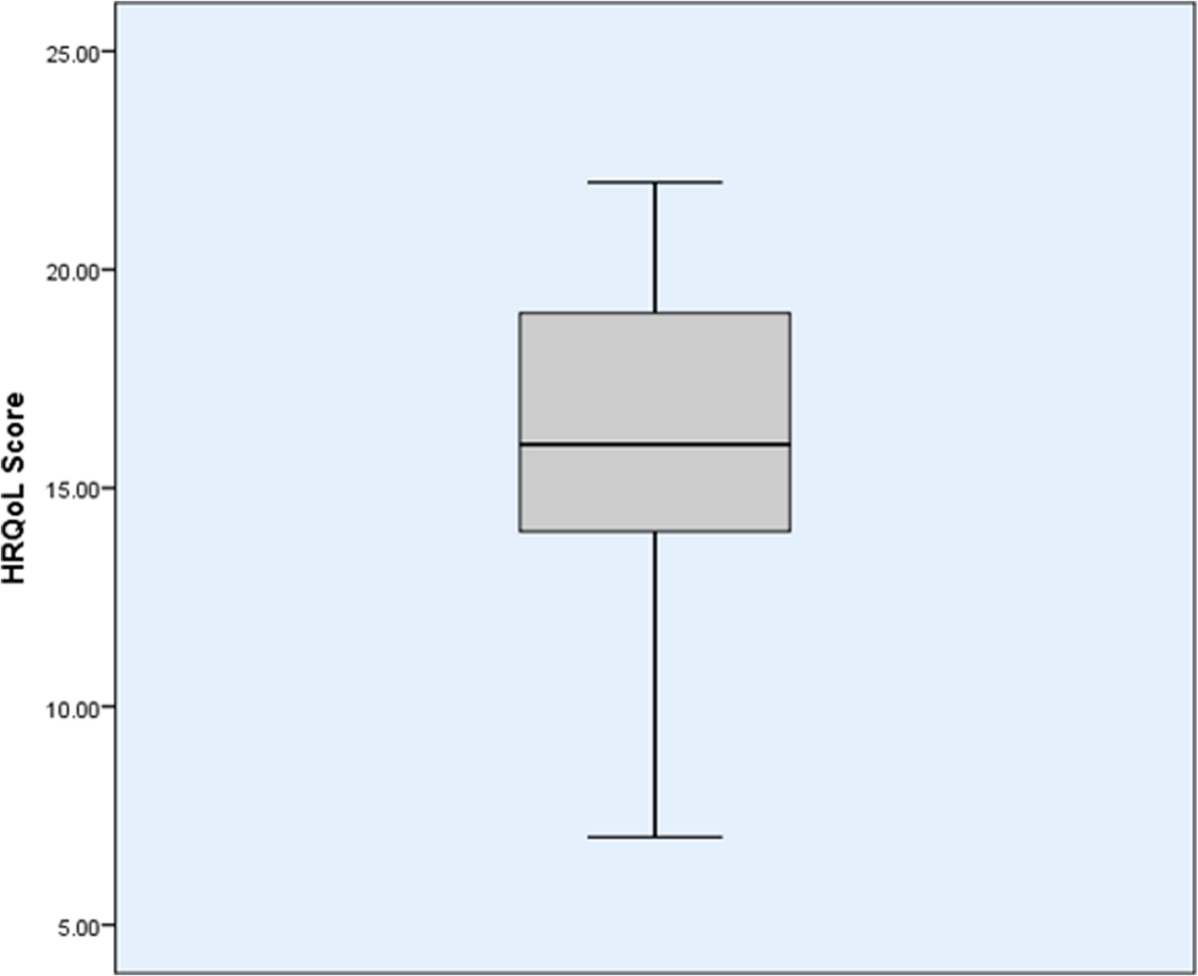
Shows the Health-related Quality of Life score of Children with thalassemia; 16 is the mean score and 50% of children are in the score of 13 to 19

**Fig. 2:**
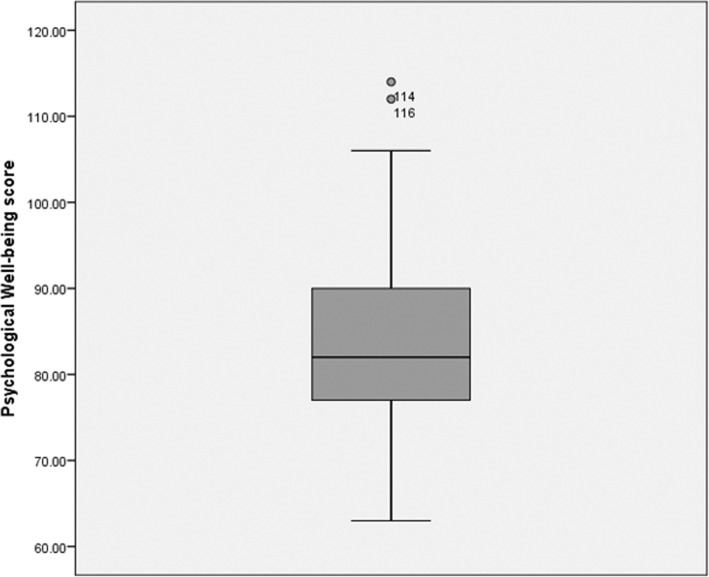
Shows the psychological well-being score of parents; 84 is the mean score and two outliers (score of 114 and 116) and 50% of parents are in the score of 77 to 90

On 125 thalassemia-affected children, 68 were boys and 57 were girls. All the children are suffered from transfusion-dependent thalassemia major. Out of the study population, 50% of the children needed at least once a month blood transfusion; 47% needed transfusion twice a month and 3% required more than twice a month. Table 1 shows the demographic characteristics of the study population. The mean age of the children and parents were 6 years and 26 years, respectively. Average HRQoL was 16.28 with a standard deviation of 3.432. Out of this, 45% of them had an above average score (Fig. 1). The mean psychological well-being score for the parent is 83.99 with a standard deviation of 11.41 (Fig. 2). Two parents had a high well-being score of 114 and 116, which are outliers in the study. Independent sample *t*-test showed no significant difference in psychological well-being score among mothers and fathers (*t* (125) = − 0.646, *p* = 0.519). The well-being score of the parents remains the same irrespective of their gender difference. Similarly, no difference in scores of HRQoL among male and female children (*t* (123) = − 0.776, *p* = 0.969). A positive correlation was found to exist between parents’ psychological well-being and children’s HRQoL (*r* = 0.329, *n* = 125, *p* < 0.001).

**Table 1:** Demographic characteristics

	Parent	Children
Gender		
Female	72 (57.6%)	57 (45.6%)
Male	53 (42.4%)	68 (55.4%)
Age (years)	26 ± 4.86	6 ± 3.67
Educational Level		
Illiterate	7 (5.6%)	
Secondary school and lower	45 (36%)	
Diploma or bachelor of science	55 (44%)	
Master of science or higher	18 (14.4%)	
Religion		
Hindu	34 (27.2%)	
Muslim	66 (52.8%)	
Christian	25 (20%)	
Monthly income (Rs.)		
< 5000	73 (58.4%)	
5000 to 15,000	42 (33.6%)	
> 15,000	10 (8%)

Multiple regression analysis was used to ascertain the factors influencing the HRQoL of thalassemia-affected children. Six predictors accounted for 37% of the variance (*R*^2^ = .37, *F* (11,113) =4.023, *p* < .01); out of which three predictors significantly provided information on children’s HRQoL (Table 2).

**Table 2:** Factors affecting the health-related quality of life of children with thalassemia

Predictors	Regression Coefficient
Child’s age	−0.043
Child’s education	1.991*
Transfusion interval	0.003
Educational qualification	1.109*
Monthly income	1.023*
Parent’s well-being	−0.236

*Level of significance at *p*-value <.05

## Discussion

In the current study, we have found certain factors that have an influence on the HRQoL thalassemia-affected children. Among them, a major factor focused in this study was the parents’ psychological well-being, which was proved to be significantly associated with children’s HRQoL. Certain factors like children’s and parent’s education status, and family’s monthly income were found to have a significant influence on children’s HRQoL.

Education is a key indicator of income, family growth, sustenance, and well-being. Educated parents bring a warm and pleasing social climate at home comparing to non-educated parents [20]. Children with educated parents are more inclined to have educational support, moral advice, economic background, nutritional support, assisted in taking right decisions, and help to face a problem with a positive attitude. Education plays a role in psychological, emotional, social well-being than the other aspects of well-being [21]. Health outcomes are also influenced by education [22]. Education makes a person have a job; income; fulfillment of needs; better well-being; better health. Likewise, family income also has a major impact on children’s life [23, 24]. Our results validate the fact that education and income have a direct influence on the children’s quality of life [25].

As child ages, his or her maturity level and self-satisfaction also increase. However, in this study, it was found that children’s age plays no role in predicting their HRQoL. The frequency of transfusion is dependent on the severity of the disease condition. But, our study results suggested that transfusion interval doesn’t influence the children’s quality of life.

Through this study, we found that factors like family income, education of parents and children may have a positive influence on the HRQOL of children with thalassemia. Our study also suggests children’s HRQoL has a positive correlation with the parent’s psychological well-being. Parent’s well-being (economic, social and psychological) has even a greater influence on a disabled child’s health [26]. Children’s quality of life is connected with parent’s well-being, as the parent remain the main pillar of support to children grow in all aspects [24, 27, 28]. Family well-being is definitely a foundation for children’s quality of life. Other influencing factors need to be explored and targeted for improving the quality of life of thalassemia patients. As this is an observational study, factors associated with HRQoL are conjectural and should be viewed in that perspective. More studies to be conducted for validation of our results.

### Strength

This study was carried out in Tamil Nadu, India, where treatment burden and social stigma are higher for thalassemia-affected children’s families. This is the first study to be done, to the best of our knowledge, in India focusing on the influence on parents’ psychological well-being on HRQoL of children with thalassemia. This study was also included participants from different districts of Tamil Nadu registered in the thalassemia treatment center, which makes this study a reflection of the state as a whole. We have reported study design, sample selection, data collection, analysis and potential bias as per the STROBE guidelines. The data were double entered and validated to ensure data quality and to avoid transcription errors. Globally, many studies had focused separately on the quality of life of thalassemic children and psychological issues in caregivers/parents of thalassemic children.

### Limitations

There could also be other possible factors influencing the quality of life of thalassemia affected children and they need to be studied. A case–control study might be more appropriate to capture the HRQoL and factors influencing it. However, due to financial and time constraints, authors only focused on the factors described above. Apart from this, this study could have had the confounding bias of patients belonging to well-off families as they could access the treatment centre. A section of lower-socioeconomic status population that has no access to the treatment centre might have been missed in the study.

## Conclusion

Family well-being is the foundation for the quality of life of children It was found that factors such as family income and parent’s and children’s education have a direct association with HRQoL of children with thalassemia. However, more studies need to be done in order to ascertain the factors contributing to HRQoL of children affected with thalassemia to improve the quality of life of thalassemia patients.

## Abbreviations

HRQoL: Health-related Quality of Life
IRB: Institutional Review Board
RPWBS: Ryff Psychological Well-Being Scale
SPSS: Statistical Package for Social Sciences
